# Body composition data show that high BMI centiles overdiagnose obesity in children aged under 6 years

**DOI:** 10.1093/ajcn/nqab421

**Published:** 2021-12-30

**Authors:** Charlotte M Wright, Tim J Cole, Mary Fewtrell, Jane E Williams, Simon Eaton, Jonathan C Wells

**Affiliations:** Department of Child Health, School of Medicine, Nursing and Dentistry, University of Glasgow, Glasgow, United Kingdom; Population Policy and Practice Research and Teaching Department, UCL Great Ormond Street Institute of Child Health, London, United Kingdom; Population Policy and Practice Research and Teaching Department, UCL Great Ormond Street Institute of Child Health, London, United Kingdom; Population Policy and Practice Research and Teaching Department, UCL Great Ormond Street Institute of Child Health, London, United Kingdom; Developmental Biology and Cancer Research and Teaching Department, UCL Great Ormond Street Institute of Child Health, London, United Kingdom; Population Policy and Practice Research and Teaching Department, UCL Great Ormond Street Institute of Child Health, London, United Kingdom

**Keywords:** obesity, definition, prevalence, child, body composition, body mass index, growth charts

## Abstract

**Background:**

Most authorities define childhood overweight/obesity as a BMI exceeding the same high centile cutoff at all ages, but it seems unlikely that true obesity prevalence (excess body fat) is constant throughout childhood.

**Objectives:**

We investigated how fat mass (FM) and lean mass (LM), adjusted for height, relate to BMI and each other across childhood, using a uniquely large database of body composition measures, estimated using gold standard methods.

**Methods:**

Cross-sectional and cohort data were collated from representative samples of healthy children aged 6 wk to 20 y and children attending obesity clinics aged 7–16 y. Body composition was measured by deuterium dilution up to age 4 y, and by either deuterium or the criterion 4-component model from 4 to 20 y. FM and LM were expressed, respectively, as fat mass index (FMI; FM/height^2^) and lean mass index (LMI; LM/height^2^).

**Results:**

There were 2367 measurements of weight, height, and body composition from 1953 individuals. Before age 6 y, the variability in FMI, LMI, and BMI was much less than after; FMI was low (mainly <8 kg/m^2^) and FMI and LMI were weakly negatively correlated. From mid-childhood, upper limits for both BMI and FMI rose, but FMI in children with BMI <91st centile still rarely exceeded 8. With increasing age, the correlation of FMI with LMI rose to 0.5–0.7, driven mainly by children with a high FMI also having a high LMI.

**Conclusions:**

Raised fat levels are much less common at younger than older ages, and young children with a high BMI centile have lower FMI than older children with the same BMI centile. Current BMI centile cutoffs thus overdiagnose obesity in younger groups. More stringent cutoffs are required for children aged <6 y, matching the WHO recommendation for 0–5 y.

## Introduction

There is understandable concern about the rising worldwide prevalence of childhood overweight and obesity (1). However, the consequences for health are less clear, partly because diagnosing childhood obesity remains surprisingly difficult. Obesity is defined as a level of excess body fat that presents a risk to health, but in practice it is diagnosed using BMI, which is much easier to measure than body fat. In adults, high BMI has been associated with adverse health outcomes (2), and cutoffs of 25 and 30 kg/m^2^ are used to define overweight and obesity, respectively, in both sexes (3). In childhood it is less practical to relate BMI to much-delayed health outcomes, and absolute cutoffs cannot be used, because the BMI distribution changes markedly as children grow. Age-specific BMI centile charts were developed in the 1990s (4–6), which used BMI centile to define childhood obesity. For example, the US CDC BMI chart uses the 95th centile to define the upper 5% of the reference population as having obesity, and this applies at all ages (4). Although these upper centiles were originally used to identify children *at risk* of future overweight or obesity (5, 6), in recent years they have increasingly been regarded as synonymous with *actual* obesity. However, the centile cutoffs reflect “age agnosticism,” which assumes that obesity risk is constant throughout infancy and childhood. It has been suggested that this approach results in overdiagnosis of obesity in younger children (7). Since the 1990s, average BMI has increased in all age groups (8), but more so in older children, suggesting that the age-agnostic approach, even if originally valid, is no longer so, and the WHO has proposed more stringent cutoffs for ages 0–5 y (9).

However, to establish the true prevalence of childhood obesity, specific measures of body fat are required. A limitation of BMI at all ages is that excess BMI could reflect high fat mass (FM) or high lean mass (LM), or both (10). Two children of the same age, sex, and BMI can have 2-fold variation in FM (11). Technological developments in recent decades have led to new ways to estimate FM, but they vary substantially in precision and accuracy and very few have been applied in large samples of children across childhood. However, in recent years the 4-component (4C) model, which combines several measurements to largely cancel out the limitations of individual methods, has been applied in large childhood studies (12). In children aged <5 y, the simpler 2-component deuterium method provides a robust alternative (12, 13). Such data make it feasible for the first time to describe how height-adjusted FM (FM index, or FMI) and LM (LM index, or LMI) vary with age, and to explore the extent to which the now conventional BMI centile–based definitions reflect elevated fatness at different ages. Using a database of body composition measures from >2500 individuals of mixed ethnicity aged from 6 wk to 20 y, we describe how the distributions and intercorrelations of BMI, FMI, and LMI vary by age and sex, and how FMI corresponds to BMI centiles at different ages. It is widely assumed that FMI correlates with BMI, justifying the use of high BMI centiles to diagnose high levels of fatness, but we hypothesized that interrelations of both FMI and LMI with BMI would vary by age, and would be weaker at younger ages. If this hypothesis were supported, the validity of a high BMI centile as a marker of high fatness would also be reduced at young ages.

## Methods

The body composition database was collated from various mainly cross-sectional studies of healthy infants, children, and adolescents aged 0.1–20 y. In order to include sufficient numbers for summary statistics in each age group, per sex, we combined studies of growth and energy metabolism, population representative samples recruited to generate body composition or lung function reference data, and methodological studies (e.g., bioelectrical impedance calibration or validation of air-displacement plethysmography). To include larger numbers of children with obesity, we also included baseline data from individuals aged 7–16 y who were participating in clinical trials of treatment for obesity, for use only to explore correlations. A list of the different studies with the period of data collection and the numbers involved are given in **Supplemental Table 1**, and the distribution by age and sex in **Table 1** (12, 14–28). Ethical approval was provided as appropriate from the Medical Research Council Dunn Nutrition Unit Ethics Committee and Cambridge Local Research Ethics Committee (studies in Cambridge), the National Health Service Research Ethics Committee (Glasgow), the ALSPAC Ethics Committee (Bristol), Great Ormond Street Hospital and UCL Institute of Child Health Ethics Committee, the National Hospital for Neurology and Neurosurgery and Institute of Neurology Joint Research Ethics Committee, and London-Hampstead Research Ethics Committee (London). In every study, parents, and participants aged ≥16 y, provided written informed consent whereas verbal assent was obtained from children and adolescents aged <16 y. In most studies, ethnic ancestry was recorded based on parental report, or on self-report for adolescents, as “white” (European ancestry), “black” (African or Caribbean ancestry), “South Asian” (ancestry from Indian subcontinent), or “other/mixed” ethnicity (25).

**TABLE 1:** Mean and SD for BMI, fat mass index, and lean mass index by age and sex^1^

			BMI, kg/m^2^		FMI, kg/m^2^		LMI, kg/m^2^	
Age, y	Sex	*n*	Mean	SD	Mean	SD	Mean	SD
0 to <3	Males	160	16.8	1.53	4.0	1.32	12.8	1.06
	Females	194	16.3	1.53	4.1	1.29	12.1	1.00
3 to <6	Males	73	16.1	1.64	3.4	1.18	12.7	1.25
	Females	72	16.2	1.96	4.1	1.52	12.0	1.12
6 to <9	Males	255	16.9	3.37	3.8	2.48	13.1	1.43
	Females	258	17.6	3.79	4.9	2.82	12.7	1.45
9 to <12	Males	269	18.5	4.38	4.9	3.34	13.7	1.62
	Females	312	20.4	4.96	6.5	3.66	13.8	1.80
12 to <15	Males	260	20.5	5.44	5.0	3.98	15.5	2.30
	Females	281	22.0	6.20	6.9	4.31	15.2	2.41
15 to <20	Males	108	22.5	4.37	4.4	3.11	18.1	2.04
	Females	125	22.5	4.31	7.0	3.18	15.6	1.56

1 FMI, fat mass index; LMI, lean mass index.

All children had weight and length (infants), or height (children) measured in duplicate using standard protocols. Body composition was measured either by total body water estimated by deuterium dilution (13), or by the 4C model, which integrates body weight, body volume, total body water, and bone mineral content (12). These 2 methods accurately partition body weight into FM and LM, which are then adjusted for height by dividing by height squared, to give the LMI and FMI in the same units as BMI (29).

### Analysis

BMI (kg/m^2^) was converted into *z*-scores compared with the UK 1990 reference (5) to group data into centile categories using the 25th, 50th, 75th, 91st, and 98th centiles (5) as cutoffs, which correspond to *z*-scores spaced two-thirds of a unit apart (30). Mirrored density (violin) plots for FMI, LMI, and BMI, excluding the clinical data, were constructed to illustrate frequency distributions by 2-y age group and sex, along with mean cubic P-spline regression curves compared with age with 95% confidence bands. Also, FMI was plotted against LMI and BMI, and LMI compared with BMI, by 3-y age group (plus 15–20 y) and sex, including the Pearson correlation and cubic P-spline regression curve and 95% confidence band for each age group.

Grouped data were summarized as mean and SD. The analysis aimed to identify differences in the relations across age groups by sex. Correlations were compared for significance across groups using the Fisher *z* transformation. GAMLSS (Generalized Additive Models for Location, Scale and Shape) was used to compare the relations between FMI, LMI, and BMI within and across groups (31). GAMLSS is a form of multiple regression that tests not only for linearity and curvature, but also for heteroscedasticity, that is, the residual error changing as the *x*-variate changes. For each age group 3 models were fitted: *1*) the linear regression; *2*) the P-spline curve, and *3*) as (*2*) but with heteroscedasticity adjusted for. The differences in deviance between models were tested for significance using χ^2^.

In addition, cutoffs for raised FMI, based on the observed distribution, were used to define overweight and obesity, respectively, and the ability of BMI-based cutoffs for overweight (+1 SD) and obesity (+2 SD) to detect such children were tested in terms of sensitivity, specificity, and positive and negative predictive value.

All analyses were carried out using R version 4.1.2 (The R Foundation), with the plots drawn using ggplot2 and patchwork. The level *P* < 0.01 was used to define statistical significance.

## Results

The database consisted of 2367 sets of measurements in 1953 individuals, of which 92 were selected via obesity clinics; 1155 individuals, with 1566 measurements, were of white ethnicity, whereas 375, 360, and 63 respectively were black, South Asian, and other/mixed ethnicity (see **Supplemental Figure 1**). The patterns described below did not differ by ethnicity, and therefore ethnicity has not been emphasized in the analysis.

**FIGURE 1:**
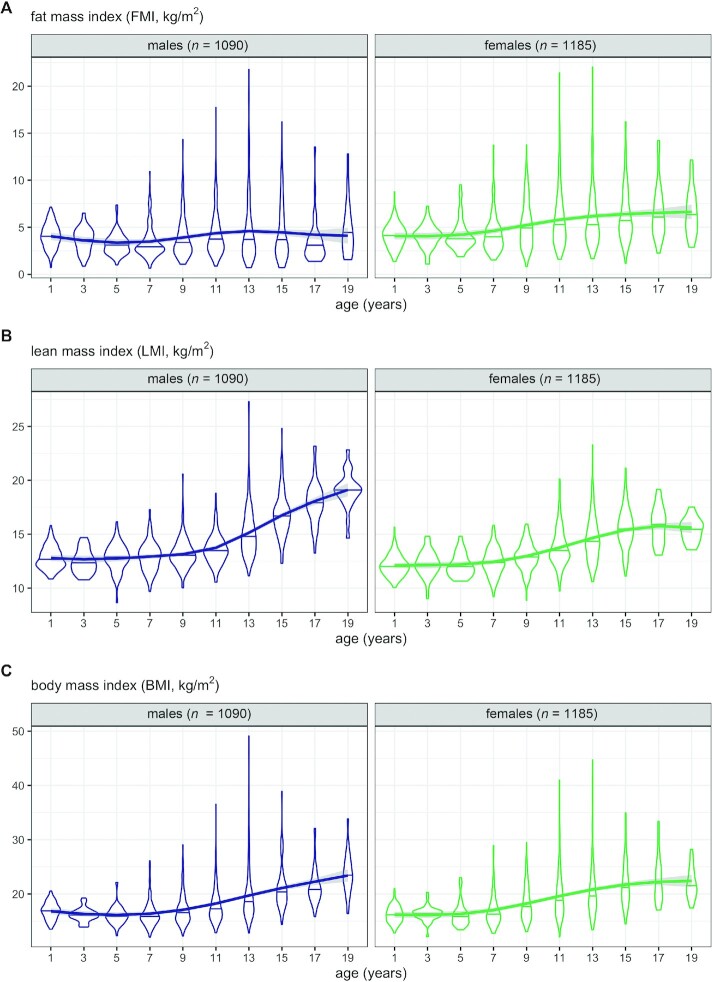
Violin plots (mirrored density plots) of (A) FMI, (B) LMI, and (C) BMI by age group and sex, excluding obesity clinic patients. The horizontal line in each “violin” indicates the median. Mean cubic P-spline regression curves with 95% confidence bands are superimposed. FMI, fat mass index; LMI, lean mass index.

Table 1 summarizes the data by sex and age group. **Figure 1** shows the frequency distributions of FMI, LMI, and BMI as violin plots by age group and sex, along with the mean curves by age. For FMI (Figure 1A) the distribution was close to normal in early life, but it became right skewed with increasing age and the skewness peaked in puberty. Note that the distance between mean curve and median line is a measure of skewness. Median and mean FMI were consistently higher in females than males, and the sexes diverged with age. For LMI (Figure 1B) there was less skewness and it emerged only later. Median and mean LMI were consistently higher in males, and again the sexes diverged with age. For BMI (Figure 1C) the 2 sexes showed similar distributions in mean, median, and skewness, and the skewness peaked in puberty. For BMI and FMI the variability increased sharply after age 6 y.

### Relations of FMI with LMI and BMI

**FIGURE 2:**
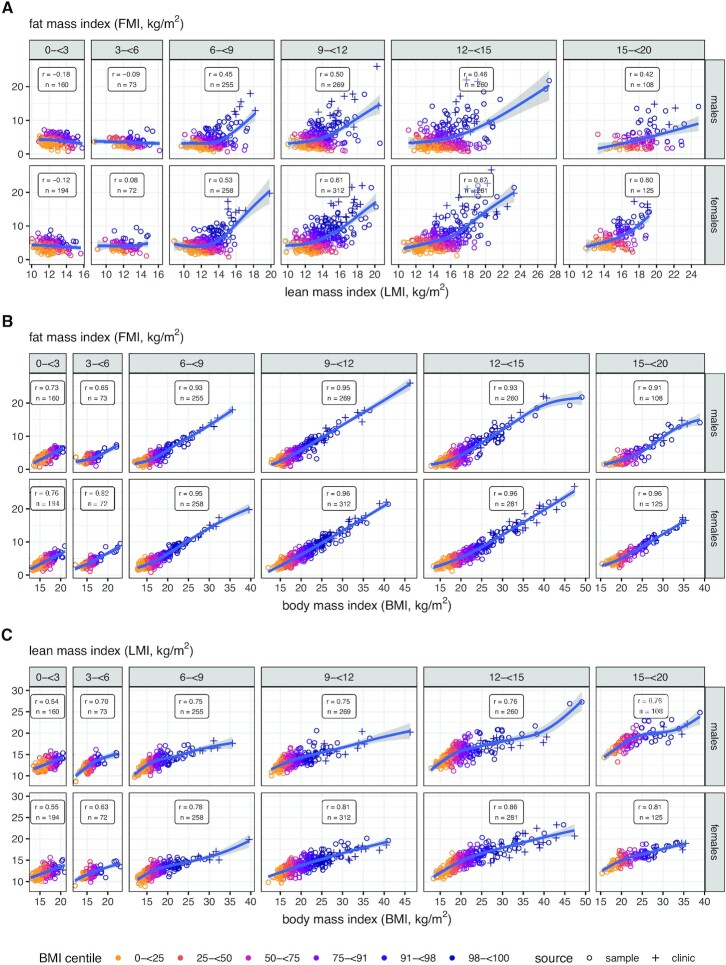
Scatterplots of (A) FMI against LMI, (B) FMI against BMI, and (C) LMI against BMI by age group and sex, each with Pearson correlation coefficient *r*, sample size *n*, cubic P-spline regression curve, and 95% confidence band. Points are color-coded by BMI centile group: yellow <25th; orange 25 to <50th; mauve 50 to <75th; purple 75 to <91st; dark purple 91 to <98th; navy blue >98th. Obesity clinic patients shown as “+.” FMI, fat mass index; LMI, lean mass index.

**Figure 2** shows correlations and trends for FMI compared with LMI and BMI, and LMI compared with BMI, by age group and sex. **Supplemental Table 2** gives the corresponding group summary statistics. For FMI compared with LMI, correlations were small, mainly negative, and statistically nonsignificant up to age 6 y, between −0.18 (*P* = 0.02) and 0.08 (*P* = 0.5) (Figure 2A), whereas after 6 y they were large, positive, and significant, from 0.42 to 0.67 (*P* < 0.001). The regression curves were also linear before 6 y (*P*_nonlinearity_ = 0.1) but markedly curved thereafter (*P* < 0.001), with a steeper slope at high LMI. Variability was also greater after 6 y (*P* < 0.001), and after 3 y there was clear evidence of heteroscedasticity, with greater variability at high LMI than low (*P* < 0.001).

Between FMI and BMI (Figure 2B) the relation was different. The correlations before 6 y ranged from 0.65 to 0.82 (*P* < 0.001) yet were still significantly weaker (*P* < 0.001) than the corresponding correlations after 6 y, which all exceeded 0.9. The regression curves were close to linear in all age groups, and they did not differ significantly in slope across groups (*P* > 0.1). The mean within-group regression coefficients by sex (in units of kg/m^2^ per kg/m^2^) were 0.62 (SE = 0.014) for males and 0.66 (SE = 0.0088) for females, adjusted for heteroscedasticity.

The correlations between LMI and BMI (Figure 2C) were midway between those for FMI-LMI (Figure 2A) and FMI-BMI (Figure 2B), and the regression curves were more curved than for FMI-BMI (Figure 2B).

**Supplemental Figure 2** shows Figure 2 further classified by white compared with other ethnic ancestry. There were no obvious differences attributable to ethnicity.

**FIGURE 3:**
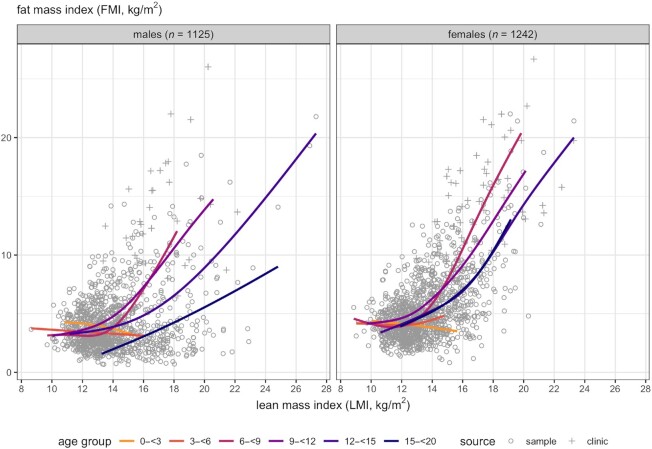
Scatterplots and cubic P-spline regression curves of FMI against LMI from Figure 2, superimposed by age group, for males and females separately. Points are color-coded by age group: yellow 0 to <3 y; orange 3 to <6; pink 6 to <9; mauve 9 to <12; purple 12 to <15; navy blue 15 to <20. Obesity clinic patients shown as “+.” FMI, fat mass index; LMI, lean mass index.

**Figure 3** superimposes the age-specific spline curves of FMI compared with LMI by sex seen in Figure 2, showing a pattern across age groups of near-horizontal lines for LMI in children aged <6 y, and also for older children with LMI below 12–13 kg/m^2^. The variability in FMI increased steeply with increasing LMI.

### Age trends in FMI and LMI by BMI centile band

**FIGURE 4:**
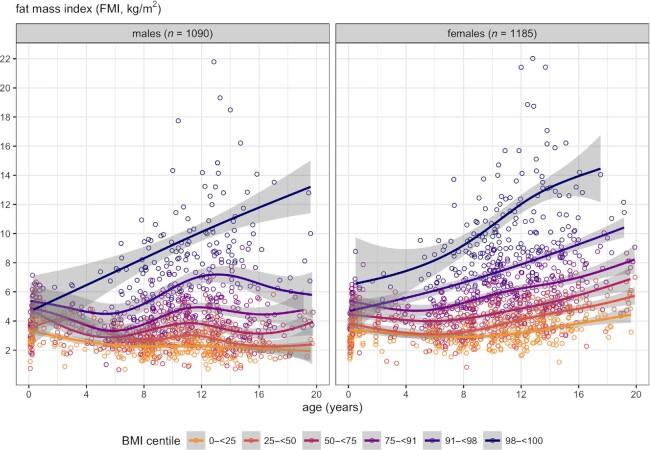
Cubic P-spline regression curves with 95% confidence bands of FMI compared with age, grouped by sex, excluding obesity clinic patients, and color-coded by BMI centile group: yellow <25th; orange 25 to <50th; mauve 50 to <75th; purple 75 to <91st; dark purple 91 to <98th; navy blue >98th. FMI, fat mass index.

**Figure 4** shows cross-sectional age trends for FMI in different BMI centile bands. The trend curves for FMI were ranked in order of BMI centile except in infancy, but the CIs for each curve overlapped up to 4 y, and the highest curves were close to the others in infancy but diverged rapidly with increasing age. The upper curves were strikingly different from the lower curves, increasing linearly from infant to adult.

### Diagnostic accuracy of raised BMI to detect raised FMI

**FIGURE 5:**
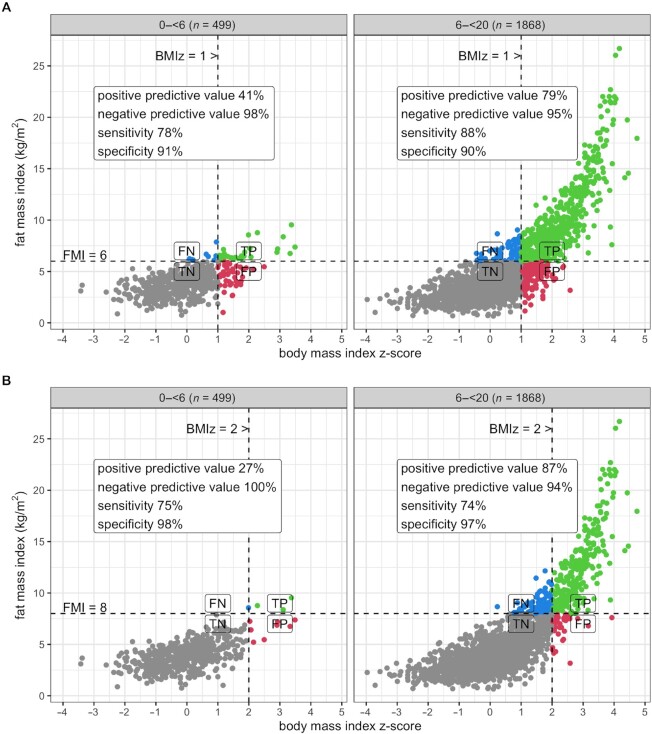
Accuracy of BMI cutoffs to detect raised and high fat mass in children aged 0 to <6 y and 6 to <20 y. (A) BMI *z*-score + 1 to detect raised fat mass (FMI >6 kg/m^2^); (B) BMI *z*-score + 2 to detect high fat mass (FMI >8 kg/m^2^). BMIz, BMI *z*-score; FMI, fat mass index; FN, false negative; FP, false positive; TN, true negative; TP, true positive.

Figure 4 demonstrates that a majority of females aged <12 y and males at all ages have FMI <6 kg/m^2^ and most are <8 kg/m^2^ at all ages. On this basis, we used cutoffs of FMI = 6 kg/m^2^ for raised FMI and FMI = 8 kg/m^2^ for high FMI, and tested the positive predictive values (PPVs) of BMI-based cutoffs for overweight (+1 SD) and obesity (+2 SD) for detecting children with raised or high FMI, for ages under and over 6 y (**Figure 5**). This revealed that less than half of younger children in the overweight BMI range also had raised FMI (PPV = 41%), compared with a majority of older children (PPV = 79%). In the obese BMI range there were few younger children and only 27% of these had high FMI, whereas many more older children were above the cutoff and 87% of them also had high FMI.

## Discussion

The limitations of BMI for assessing childhood obesity are well recognized (10), and in older children and adolescents alternative approaches have been proposed, including centiles for percentage body fat (32) or FMI (12), or the revision of the calculation of BMI to improve its association with adiposity (33). FMI reference centiles are also available for infants and children aged 6 wk to 5 y (13). Nevertheless, outside research settings, BMI remains the primary approach for obesity assessment in children, and no previous study has addressed the question we focus on here, namely whether it is appropriate to use a fixed BMI centile to categorize obesity across the entire pediatric age range.

Our analysis was based on a uniquely large database of body composition from infancy to adulthood, measured using gold standard 4C or isotope methodology. These data encompass a wide range of BMI, throughout childhood, with greater variability in mid-childhood, partly due to the contribution of children from obesity clinics. The data allowed us to describe in detail how BMI, FMI, and LMI vary by age and sex, and with each other, and the figures illustrate visually what a numerical summary alone cannot. The obesity clinics provided important extra data about children with BMI above the normal range—the group of most concern—who are necessarily rare in normative samples. Previous analyses of these data (14) showed that FMI is lower than indicated by earlier studies based on single, indirect methods (34, 35). This expanded dataset reveals that in young children FMI was generally low, whereas from mid-childhood onwards, more children had raised FMI and the upper limits for both BMI and FMI rose.

The intercorrelations of BMI, FMI, and LMI reveal a complex, triangular relation between weight, FM, and LM that varies strikingly with age. Up to 6 y the distribution of FMI and LMI, as well as the relation between them, is very different compared with later ages. It has already been shown that BMI is a poor marker of adiposity in individual children, being confounded by variability in LMI (11). What this analysis adds is that the age-agnostic assumption that high BMI is uniformly associated with high adiposity across the pediatric age range is also flawed, because the correlation between FMI and BMI was mainly negative and also statistically nonsignificant before 6 y, whereas it was consistently large, positive, and significant after 6 y.

A similar age-based discrepancy in the relation between BMI and percentage body fat measured by DXA was observed in a previous study (36), but it was assumed by the authors to reflect a problem with the percentage fat cutoffs. This lack of a significant association of BMI with fatness in young children would help explain why overweight tracks so weakly from younger ages to later childhood (7, 37, 38), and why there is no association of high BMI in children aged <6 y with adult noncommunicable disease risk (39).

This striking difference between younger and older children in the association of high BMI with high fatness provides support for the recent suggestion that obesity is actually rare in young children (7) and that the approach to obesity categorization might need to be fundamentally revised.

Ideally, the diagnosis of obesity should rely on the direct measurement of FM, rather than BMI as its proxy. Our results suggest that this should be based on FM adjusted just for size (FMI) rather than FMI *z*-score. This is because the *z*-score adjusts for the change in variability with age, and so masks the greatly increased proportion of older children with raised FMI. Based on the data in Figures 2A and 4, it would be reasonable to use 6 kg/m^2^ as an FMI-based cutoff for overweight and 8 kg/m^2^ as a cutoff for obesity, for ages up to at least the early teens.

Until accurate methods for routine assessment of FM in children become available, BMI cutoffs for overweight and obesity will still be needed. The prevalence of high FMI at younger ages was too low to undertake a Receiver Operator Characteristic (ROC) assessment, but it is clear from Figure 5 that there is poor concordance with high BMI. This suggests the need to adopt the approach proposed 10 y ago by the WHO (9). This defines overweight and obesity cutoffs as +2 and +3 BMI WHO *z*-scores, respectively, for children aged <5 y, but +1 and +2 *z*-scores, respectively, for children aged 5–19 y (9). The effect of this would be to greatly reduce the prevalence of obesity in preschool children, shifting attention to older children and teenagers, who have levels of body fat that are more likely to be genuinely harmful.

Our analyses also reveal BMI-dependent associations between LMI and FMI in older children. Whereas FMI and LMI tended still to be uncorrelated for children with BMI below the 91st centile, LMI and FMI were quite strongly correlated at higher BMI centiles, the most extreme being children recruited from obesity clinics. This association of high LMI with high FMI is consistent with earlier case-control studies (40). In children with obesity, increased FMI might be expected to drive increased muscle mass or even bone size, in response to the load-carrying requirements of the extra fat stores. Alternatively, it has been suggested that LMI could itself play a role in obesity development. Potential underlying mechanisms remain uncertain, but the protein-leverage hypothesis suggests that children with high LMI (and higher protein requirements) tend to overconsume energy if exposed to a low-protein highly processed diet (41).

The study inevitably has limitations. The sampling was not all population representative because the data in mid-childhood included children oversampled for obesity, though their data were shown separately in Figure 2 and excluded from Figures 1 and 4. The data on the youngest children also dated from a period much earlier in the obesity epidemic, when childhood obesity was less common. Thus, we cannot estimate absolute prevalence at different ages. However, Figure 4 allows comparison of FMI stratified by BMI category, showing that before 6 y, even children with the highest BMI for age tended to have only slightly raised FMI.

The younger children were measured by deuterium, not the 4C method used at older ages, but the 2 methods show high agreement, because water is the primary component of LM (12, 14). Although the database was multiethnic, the nonwhite data were too few to analyze by individual ethnic group. However, Supplemental Figure 2 suggests that ethnic differences were small. Ethnic differences in FM and LM have been reported previously (42, 43) but, although genetic ancestry might contribute (44), variability in living conditions or societal stresses including racism will also be relevant (45). Accepting such ethnic variability, our study found that the inconsistent association of high fatness and high BMI was apparent in both white and nonwhite groups. Finally, there is a lack of data on health outcomes, whereby the immediate or downstream biological consequences of high adiposity might be assessed.

In conclusion, children with raised BMI before the age of 6 y have relatively low fat levels, and most do not actually have obesity, suggesting that centile cutoffs defining obesity in children aged <6 y need to be set higher than for older children. Research into the role of LM in the development of obesity in older children is needed.

## Supplementary Material

nqab421_Supplemental_File

## Data Availability

The data described in the manuscript, code book, and analytic code will be made available upon reasonable request to Jonathan Wells and Tim Cole. There is no 3rd party material included.

## Notes

We gratefully acknowledge funding for the original data collection from the Medical Research Council, the Wellcome Trust, the Child Growth Foundation, the British Heart Foundation, and the Scottish Government Chief Scientist Office. This research was supported by the National Institute for Health Research (NIHR) Great Ormond Street Hospital Biomedical Research Centre. The views expressed are those of the authors and not necessarily those of the National Health Service (NHS), the NIHR, or the UK Department of Health. Funders played no role in the conceptualization, methodology, data curation, analysis, or writing up of the results.

Supplemental Tables 1 and 2 and Supplemental Figures 1 and 2 are available from the “Supplementary data” link in the online posting of the article and from the same link in the online table of contents at https://academic.oup.com/ajcn/.

Abbreviations used: FM, fat mass; FMI, fat mass index; GAMLSS, Generalized Additive Model for Location, Scale and Shape; LM, lean mass; LMI, lean mass index; PPV, positive predictive value; 4C, 4-component model.
