# Supplementary material for: Body composition data show that high BMI centiles overdiagnose obesity in children aged under 6 years
Source: Am J Clin Nutr. 2021 Dec 30;116(1):122–31. doi: 10.1093/ajcn/nqab421 (PMC9257461; doi:10.1093/ajcn/nqab421)

Body composition data show that high body mass index centiles over-diagnose obesity in children aged under 6 years

Charlotte M Wright et al

Online Supplementary Material

Contents

Supplementary Table 1. Body composition data sources. 2

Supplementary Table 2. Linear regression results for Figure 2 by sex and age group. 3

Supplementary Figure 1: Flow diagram 4

Supplementary Figure 2: FMI plotted against LMI and BMI by age group, sex, and ethnicity, each with correlation coefficient, summary cubic spline curve and 95% confidence interval. Obesity clinic participants are shown with '+'. 5

Supplementary Table 1. Body composition data sources.

Study (citation)	Period	Age range	Location	% male	Method used	Number of subjects	Number of measures	Number of measures per subject
Infant energy metabolism 1 (16)	1987-1988	1.5m – 9m	Cambridge	37.5	D_2O	51	157	1-4 (median 3)
Infant energy metabolism 2 (17, 18)	1992-1995	3m – 3y	Cambridge	50.0	D_2O	42	58	1-2
Infant energy metabolism 3 (19, 20)	1995-1997	9m – 2y	Cambridge	49.0	D_2O	35	53	1-2
Infant energy-metabolism 4 (21)	2007-2008	4 – 6m	Glasgow	50.0	D_2O	28	56	2
Child energy metabolism (22)	1991-1992	1.5 – 4.5y	Cambridge	51.9	D_2O	77	77	1
DXA validation study (23)	1995-1996	5 – 11y	Cambridge	51.2	D_2O	41	41	1
Young 4C methods study (24)	2001-2002	4 – 7y	Cambridge	50.0	4C	20	20	1
DXA calibration study (15)	2000	8 – 9y	Bristol	50.0	D_2O	50	50	1
Older 4C methods study (14)	1996-1997	8 – 12y	Cambridge	53.3	4C	30	30	1
Reference child (12)	2001-2016	4 – 20 y	London	49.4	4C	509	755	1-5 (median 1)
BIA validation children (25)	1995-1996	5 – 11y	London	47.5	D_2O	598	598	1
BIA calibration adolescents (26)	2008-2009	11 – 15y	London	47.1	D_2O	380	380	1
Obesity clinics (27, 28)	2003-2008	6 – 19y	London	38.0	4C	92	92	1

Age: m – months, y – years; Method: D_2O – deuterium dilution, 4C – 4-component model

Supplementary Table 2. Linear regression results for Figure 2 by sex and age group.

Sex	Age group (years)	Correlation coefficient + 95% CI	Regression coefficient + 95% CI
		**FMI-LMI**	
boys	0-<3	-0.18 (-0.33, -0.03)	-0.23 (-0.43, -0.03)
girls	0-<3	-0.12 (-0.26, 0.03)	-0.15 (-0.33, 0.03)
boys	3-<6	-0.09 (-0.32, 0.15)	-0.08 (-0.30, 0.14)
girls	3-<6	0.08 (-0.16, 0.31)	0.11 (-0.21, 0.43)
boys	6-<9	0.44 (0.34, 0.54)	0.77 (0.57, 0.97)
girls	6-<9	0.53 (0.44, 0.62)	1.03 (0.83, 1.23)
boys	9-<12	0.5 (0.40, 0.59)	1.03 (0.81, 1.25)
girls	9-<12	0.61 (0.53, 0.67)	1.24 (1.06, 1.42)
boys	12-<15	0.46 (0.36, 0.55)	0.79 (0.59, 0.99)
girls	12-<15	0.68 (0.60, 0.74)	1.21 (1.05, 1.37)
boys	15-<20	0.42 (0.24, 0.56)	0.64 (0.36, 0.92)
girls	15-<20	0.6 (0.48, 0.70)	1.23 (0.93, 1.53)
		**FMI-BMI**	
boys	0-<3	0.73 (0.65, 0.80)	0.63 (0.53, 0.73)
girls	0-<3	0.76 (0.70, 0.82)	0.64 (0.56, 0.72)
boys	3-<6	0.65 (0.49, 0.77)	0.47 (0.35, 0.59)
girls	3-<6	0.82 (0.73, 0.89)	0.64 (0.54, 0.74)
boys	6-<9	0.92 (0.90, 0.94)	0.68 (0.64, 0.72)
girls	6-<9	0.95 (0.93, 0.96)	0.70 (0.68, 0.72)
boys	9-<12	0.95 (0.93, 0.96)	0.72 (0.70, 0.74)
girls	9-<12	0.96 (0.95, 0.97)	0.71 (0.69, 0.73)
boys	12-<15	0.93 (0.91, 0.94)	0.68 (0.64, 0.72)
girls	12-<15	0.96 (0.95, 0.97)	0.67 (0.65, 0.69)
boys	15-<20	0.91 (0.86, 0.94)	0.64 (0.58, 0.70)
girls	15-<20	0.96 (0.94, 0.97)	0.71 (0.67, 0.75)
		**LMI-BMI**	
boys	0-<3	0.54 (0.41, 0.64)	0.37 (0.27, 0.47)
girls	0-<3	0.55 (0.44, 0.64)	0.36 (0.28, 0.44)
boys	3-<6	0.7 (0.55, 0.80)	0.53 (0.41, 0.65)
girls	3-<6	0.63 (0.47, 0.76)	0.36 (0.26, 0.46)
boys	6-<9	0.75 (0.69, 0.80)	0.32 (0.28, 0.36)
girls	6-<9	0.78 (0.72, 0.82)	0.30 (0.28, 0.32)
boys	9-<12	0.75 (0.69, 0.80)	0.28 (0.26, 0.30)
girls	9-<12	0.81 (0.77, 0.85)	0.29 (0.27, 0.31)
boys	12-<15	0.76 (0.70, 0.81)	0.32 (0.28, 0.36)
girls	12-<15	0.86 (0.82, 0.89)	0.33 (0.31, 0.35)
boys	15-<20	0.76 (0.67, 0.83)	0.36 (0.30, 0.42)
girls	15-<20	0.81 (0.74, 0.86)	0.29 (0.25, 0.33)

Supplementary Figure 1: Flow diagram

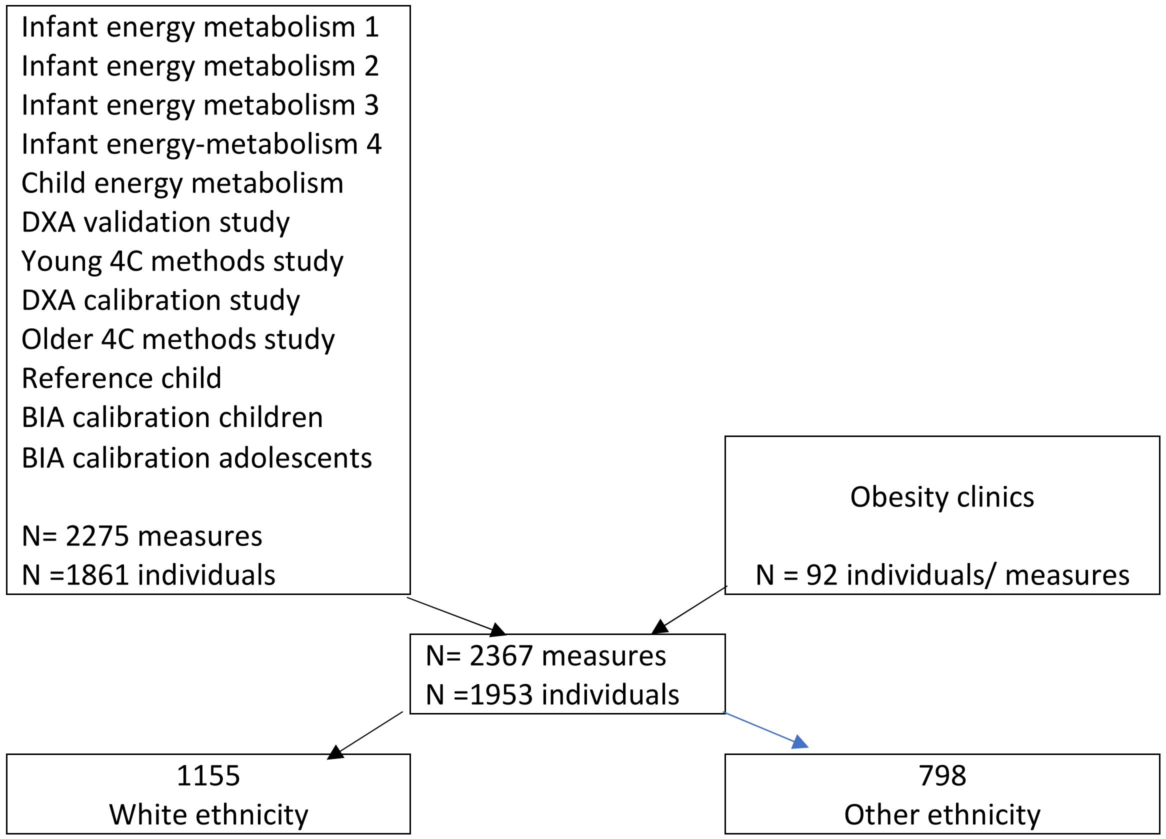

Supplementary Figure 2: Scatterplots of A) FMI against LMI, B) FMI against BMI and C) LMI against BMI by age group, ethnicity and sex, each with Pearson correlation coefficient r, sample size n, cubic P-spline regression curve and 95% confidence band. Points are color-coded by BMI centile group: yellow <25th, orange 25<50th, mauve 50<75th, purple 75<91st, dark purple 91<98th, navy blue >98th: obesity clinic patients shown as '+'.

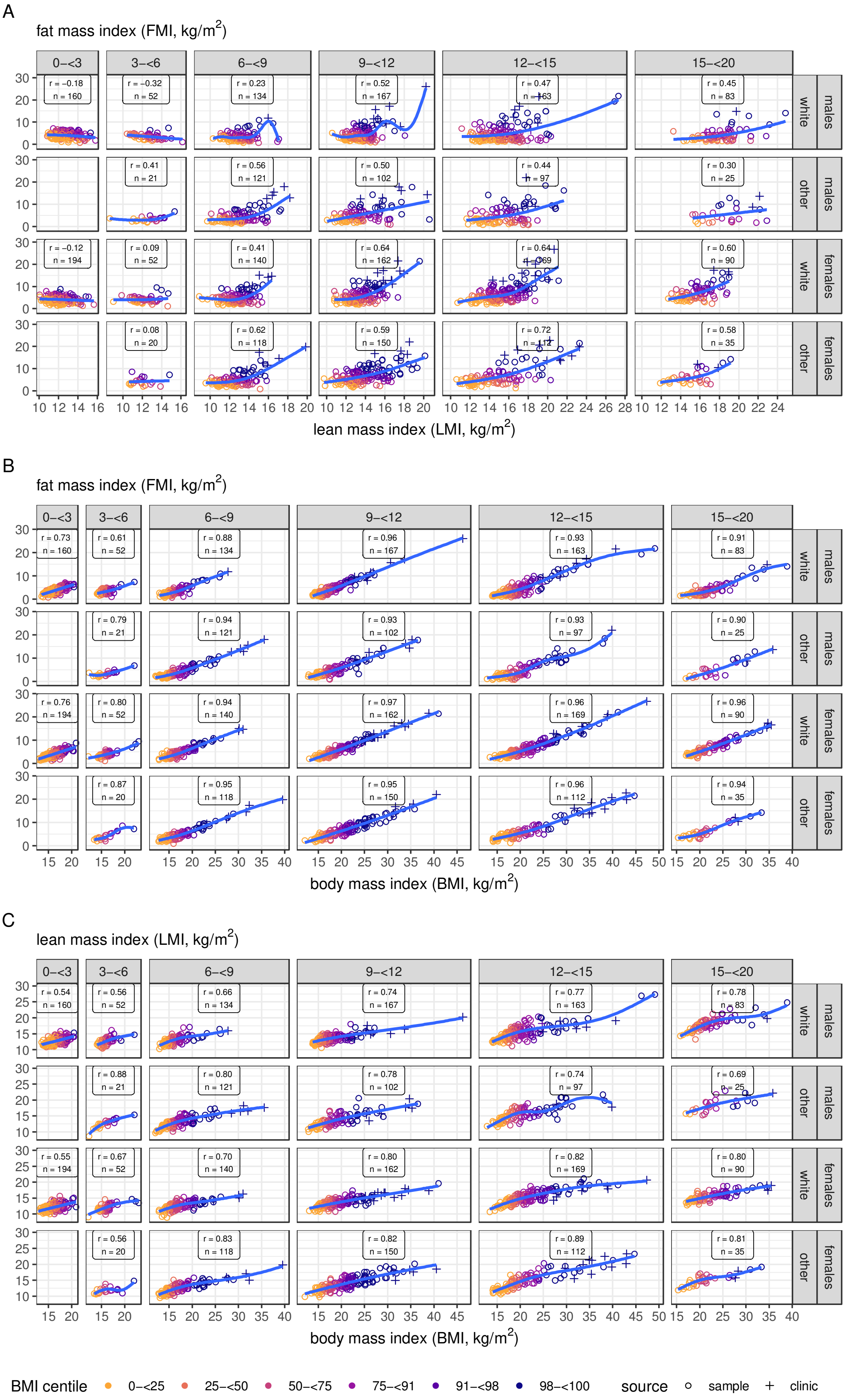

Supplement: nqab421_Supplemental_File [file nqab421_supplemental_file.pdf]
